# Fibrinous anterior uveitis following laser *in situ* keratomileusis

**DOI:** 10.4103/0301-4738.53064

**Published:** 2009

**Authors:** Pragya Parmar, Amjad Salman, M Rajmohan, Nelson CA Jesudasan

**Affiliations:** Institute of Ophthalmology, Joseph Eye Hospital, Tiruchirapalli - 620 001, Tamil Nadu, India

**Keywords:** Laser *in situ* keratomileusis, uveitis, Severe anterior uveitis

## Abstract

A 29-year-old woman who underwent laser *in situ* keratomileusis (LASIK) for myopic astigmatism in both eyes presented with severe pain, photophobia and decreased visual acuity in the left eye eight days after surgery. Examination revealed severe anterior uveitis with fibrinous exudates in the anterior chamber, flap edema and epithelial bullae. Laboratory investigations for uveitis were negative and the patient required systemic and intensive topical steroids with cycloplegics to control the inflammation. This case demonstrates that severe anterior uveitis may develop after LASIK and needs prompt and vigorous management for resolution.

Laser *in situ* keratomileusis (LASIK) is currently the most popular method for the correction of low to moderate ametropia. Information about anterior segment inflammatory changes after LASIK is limited. While anterior uveitis has been described as a complication of LASIK with an incidence of 0.18%,[1] most reported cases so far have been those of mild to moderate uveitis that respond well to topical medication.[1,2] We report a patient who developed severe anterior uveitis with fibrinous exudates in one eye eight days after undergoing LASIK for low myopic astigmatism in both eyes.

## Case Report

A 29-year-old woman underwent uncomplicated LASIK in both eyes for low myopic astigmatism. Prior systemic and ocular history was unremarkable. Preoperative refractive error was −3.00 diopter sphere (D Sph) / −1.00 diopter cylinder (D Cyl) × 180° in the right eye and −2.75 D Sph/ −1.00 D Cyl × 180° in the left eye. On her scheduled postoperative visits on Day 1 and 5, examination of both eyes revealed a clear cornea and a normal anterior segment with an uncorrected visual acuity of 20/20.

Eight days after LASIK, the patient presented with severe pain, photophobia and decreased visual acuity in the left eye. Visual acuity at this time was 20/20 in the right eye and 20/80 in the left eye. Right eye examination was normal but the left eye showed mild flap edema, few peripheral epithelial bullae with intense flare and cells in the anterior chamber with thick fibrinous exudates in the anterior chamber. There were pigments on the endothelium and the anterior lens capsule but no keratic precipitates and the corneal stromal bed was clear. The intraocular pressure (IOP) was 11 mm Hg in the right eye and 9 mm Hg in the left eye. Posterior segment examination was normal in both eyes.

**Figure 1:**
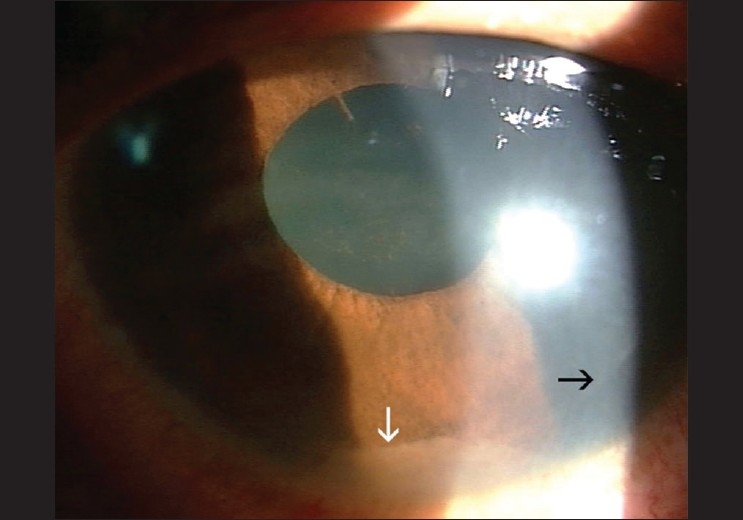
Appearance of the eye two days after starting topical steroids and cycloplegics. Inflammation persisted with a fibrinous exudate in the anterior chamber (white arrow) and early posterior synechiae superiorly. The edge of the LASIK flap is seen (black arrow)

**Figure 2:**
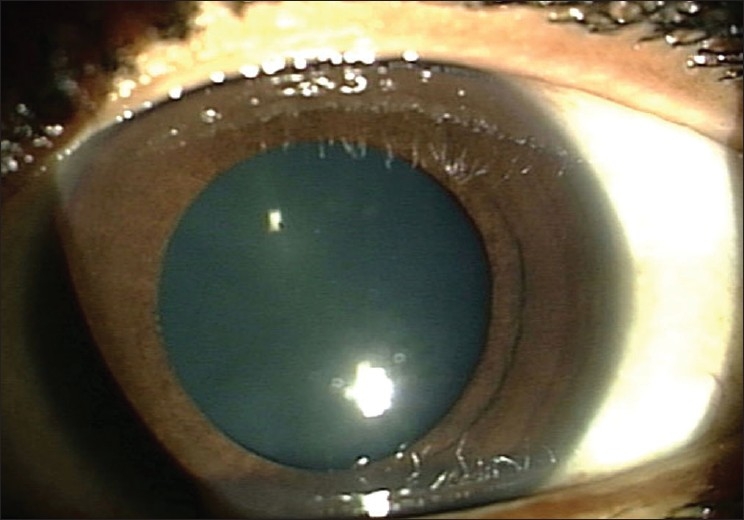
After two weeks of systemic and topical steroids, the inflammation has completely resolved

The patient was started on hourly prednisolone acetate 1% eye drops (Predmet^®^ eye drops, Sun Pharmaceuticals, India) and cyclopentolate 1% eye drops three times daily (Cyclate eye drops^®^, Cadila Pharamceuticals, India). As systemic examination by an internist proved unremarkable, a routine laboratory screen currently used at our hospital for patients with severe or recurrent anterior uveitis was ordered. This included blood counts, erythrocyte sedimentation rate (ESR), c-reactive protein, Mantoux test, venereal disease research laboratory test (VDRL), chest X-ray, human immunodeficiency virus (HIV) serology and human leukocyte antigen (HLA) typing. These investigations were within normal limits and the patient was HLA B27-negative. As the patient failed to respond adequately after two days of topical steroid therapy with persistence of the uveitis, increase in fibrinous exudates and development of early posterior synechiae [Fig. 1], oral prednisolone 1 mg/kg body weight (Tab Wysolone^®^, Wyeth Pharmaceuticals, India, 60 mg once daily) was added. The uveitis responded after starting oral steroids and gradually resolved over two weeks. At the end of two weeks, the uveitis had resolved with resolution of the flap edema and return of visual acuity to 20/20 [Fig. 2]. Oral steroids were tapered and stopped over four weeks. Intraocular pressures remained normal throughout.

## Discussion

Opinions vary regarding the effect of LASIK on anterior chamber inflammatory response with some authors[3,4] reporting an altered blood-aqueous barrier and increase in aqueous flare using a laser flare meter while others have found no such changes.[5] In one large retrospective series,[1] the annual incidence of uveitis following LASIK was found to be 0.06%. Moshirfar *et al*.[6] found no difference in the incidence of uveitis in eyes undergoing LASIK and in the fellow eyes without LASIK among HLA B27-positive patients.

Several hypotheses have been put forward to explain uveitis after LASIK including the sudden increase and decrease of intraocular pressure following application of the suction ring which simulates the effects following a closed globe injury.[7] Another theory postulates that the shock wave following each excimer application incites inflammation and that the depth of ablation correlates with the inflammatory response.[4] This relation between ablation depth and inflammatory response has not been demonstrated by other authors.[1] Our patient developed uveitis in one eye following ablation for nearly identical refractive error in both eyes suggesting that depth of ablation and number of laser spots do not correlate with the amount of inflammation.

Nearly all the uveitis following LASIK reported in literature has been of the mild to moderate variety which responds rapidly to topical medication. Our patient developed a severe anterior uveitis with fibrinous exudates that needed systemic steroids for control of inflammation. This form of uveitis has not been reported earlier. The cause for this severe inflammation in our patient is unclear. We perform LASIK in about 750 eyes annually and have not encountered any other case with anterior uveitis following LASIK. We believe the uveitis was related to the LASIK procedure as it developed within a short while of the surgery with no significant history or systemic features and extensive laboratory investigations were normal. The patient was also negative for HLA B27 which is one of the frequent associations of fibrinous anterior uveitis. It is possible that the uveitis in our patient could have been due to some other cause, such as a viral infection, which may have been missed during our lab screening. We currently encounter about 250-300 cases of uveitis annually at our hospital and in about 65% of cases of anterior uveitis, the etiology remains unclear.

In our patient, the flap edema developed despite normal IOP suggesting endothelial dysfunction associated with the severe inflammation and pigment deposition. While endothelitis is not usual in anterior uveitis, flap edema and interface fluid have been reported[2] as a cause of decreased visual acuity following uveitis. We believe that this could be due to a propensity for accumulation of fluid at the flap interface with flap edema following uveitis in eyes that have undergone LASIK. In our patient, the stromal bed remained clear and the flap edema cleared well following resolution of the uveitis.

In conclusion, severe fibrinous anterior uveitis may develop following LASIK and requires intensive treatment for resolution. LASIK surgeons should be aware of this potential complication.

## References

[1] Suarez E, Torres F, Vieira JC, Ramirez E, Arevalo JF (2002). Anterior uveitis after laser *in situ* keratomileusis. J Cataract Refract Surg.

[2] McLeod SD, Mather R, Hwang DG, Margolis TP (2005). Uveitis-associated flap edema and lamellar interface fluid collection after LASIK. Am J Ophthalmol.

[3] El-Harazi SM, Chuang AZ, Yee RW (2001). Assessment of anterior chamber flare and cells following laser *in situ* keratomileusis. J Cataract Refract Surg.

[4] Pisella PJ, Albou-Ganem C, Bourges JL, Debbasch C, Limon S (1999). Evaluation of anterior chamber inflammation after corneal refractive surgery. Cornea.

[5] Perez-Santonja JJ, Sakla HF, Cardona C, Chipont E, Alió JL (1998). Subclinical inflammation after laser *in situ* keratomileusis. J Cataract Refract Surg.

[6] Moshirfar M, Siddharthan KS, Meyer JJ, Espandar L, Wolsey DH, Vitale AT (2008). Risk for uveitis after laser *in situ* keratomileusis in patients positive for human leukocyte antigen-B27. J Cataract Refract Surg.

[7] Arevalo JF, Freeman WR, Gomez L (2001). Retina and vitreous pathology after laser-assisted *in situ* keratomileusis: Is there a cause-effect relationship?. Ophthalmology.

